# Knowledge and attitude toward intimate partner violence among couples: a baseline findings from cluster randomized controlled trial in rural Ethiopia

**DOI:** 10.3389/fpubh.2024.1467299

**Published:** 2024-11-20

**Authors:** Zeleke Dutamo Agde, Jeanette H. Magnus, Nega Assefa, Muluemebet Abera Wordofa

**Affiliations:** ^1^Department of Population and Family Health, Institute of Health, Jimma University, Jimma, Ethiopia; ^2^Department of Reproductive Health, College of Medicine and Health Sciences, Wachemo University, Hosaina, Ethiopia; ^3^Faculty of Medicine, University of Oslo, Oslo, Norway; ^4^College of Health and Medical Sciences, Haramaya University, Harar, Ethiopia

**Keywords:** attitude, Central Ethiopia, couples, intimate partner violence, knowledge

## Abstract

**Background:**

Intimate partner violence (IPV) continues to be a major public health issue in Ethiopia. Studies have shown that knowledge and attitudes play a crucial role in predicting IPV. There is a lack of comprehensive evidence on the extent and factors associated with knowledge and attitudes toward IPV in many developing countries, particularly in rural Ethiopia. The objective of this study was to assess the level of knowledge and attitudes toward IPV and associated factors among couples in Central Ethiopia.

**Methods:**

A baseline survey involving 432 couples (432 pregnant women and 432 husbands) was conducted in July, 2023, in Hadiya Zone, Central Ethiopia. Data were collected using face-to-face interviewer-administered questionnaires. Multivariable logistic regression analysis was performed, and adjusted odds ratios with 95% confidence intervals were reported.

**Results:**

The findings revealed that 53.0% of women and 58.4% of men had good knowledge of IPV, while 56.0% of women and 65.6% of men held supportive attitudes toward IPV. Women’s knowledge of IPV was significantly associated with age (25–34 years), educational attainment (secondary and higher/college education), antenatal care (ANC) visits, and husbands’ alcohol consumption. Men’s knowledge of IPV was associated with age at marriage <20, educational attainment (secondary and college/ higher education), monogamous marriage, and alcohol consumption. Predictors of women’s attitudes toward IPV included marrying before the age of 20, partner smoking, and poor knowledge of IPV. Moreover, predictors of men’s attitudes toward IPV included younger age (15–24), monogamous marriage, alcohol consumption, and poor knowledge of IPV.

**Conclusion and recommendation:**

The study found that more than half of couples had good knowledge of IPV, with a notable percentage also holding supportive attitudes toward it. Enhancing women’s education, changing attitudes, ANC visits, and addressing male substance use would help in increasing couples’ awareness of IPV and its consequences.

## Introduction

Intimate partner violence is a global public health problem, defined as any act in an intimate relationship that causes physical, psychological, or sexual harm (1). It disproportionately affects women, with it being the most common form of violence against women (2). Intimate partner violence not only violates human rights, but also raises serious public health problems (3, 4). Intimate partner violence is a universal phenomenon; it occurs in all settings and among all socioeconomic, religious, and cultural groups (5). In 2018, the World Health Organization (WHO) estimated that approximately 27% of ever-partnered women of reproductive age (6–38) globally, or over 641 million women, have experienced physical or sexual IPV at least once in their lifetimes. An estimated 33% of women in Sub-Saharan Africa are affected, which is a particularly alarming proportion (39).

Intimate partner violence is associated with serious physical, reproductive, and psychological consequences, including injuries (40), sexually transmitted infections (41), and unintended pregnancies (42). The psychological sequelae of IPV are equally profound, as IPV victims experience high rates of post-traumatic stress disorder (PTSD), depression, anxiety, and suicidal ideation (43–45). The consequences of IPV increase the cost on public health by creating long-term impacts on social functioning and mental health and placing significant strain on healthcare systems around the globe (46).

In many African societies, violence serves as a means for men to exert control over their partners, reflecting power imbalances (47). The acceptance of IPV by both men and women is a crucial indicator of its prevalence (6). Evidence from 30 countries in Sub-Saharan Africa has found that 44% of women and 25% of men accept wife beating in certain circumstances (7). According to a systematic review and meta-analysis report in Ethiopia, a substantial number (57%) of women have a supportive attitude toward IPV (8).

Pregnancy introduces additional risks, as IPV during this period has been linked to adverse maternal and fetal outcomes (9–12). The consequences of IPV contribute to increased healthcare costs and are linked to higher rates of maternal and perinatal morbidity and mortality (11, 13–17, 39).

Several socio-demographic and behavioral factors influence individuals’ knowledge of and attitudes toward IPV. Lower education levels and employment status correlate with knowledge of IPV (18, 19), while predictors of supportive attitudes include younger age, lower education, unemployment, rural residence, polygamy, lower participation in household decision-making, exposure to mass media, alcohol consumption, and poverty (7, 18, 20, 21).

Awareness and understanding of IPV are crucial in addressing and preventing its occurrence (22). Despite the recognized role of knowledge and attitude as predictors of IPV (18, 19, 21, 23, 47), there is limited evidence regarding the factors that influence these aspects within rural Ethiopian contexts. Understanding the knowledge, attitudes, and factors influencing IPV is essential for designing culturally sensitive interventions that raise awareness about its health, social, and economic impacts, address specific cultural beliefs and norms related to IPV, and promote healthier, non-violent relationships. Therefore, this study aimed to identify the level of knowledge and attitudes toward IPV and associated factors among couples in rural Ethiopia. We hypothesize that demographic and behavioral characteristics influence IPV knowledge and attitudes among women and men in the study setting.

## Methods

### Study area and period

This analysis is based on the baseline assessment for a larger study that aims to evaluate the effectiveness of couple-based violence prevention education in reducing IPV during pregnancy (24). The study was conducted in the Hadiya Zone, located in Central Ethiopia. More information about the study area (Hadiya Zone) can be found in the published study protocol (24). The study was conducted from July 1 to 30, 2023.

### Study participants’ recruitment and enrolment

The study recruited married couples, specifically pregnant women in their first trimester (less than 13 weeks) and their husbands. Eligible couples included wives within the reproductive age range (15–49 years) and husbands aged 15–60 years. Health Extension Workers (HEWs) logbook review and pre-survey were conducted to identify eligible women who were pregnant and had at least one previous live birth. A pregnancy screening questionnaire, adapted from Nega et al. (25) was used to confirm eligibility. All eligible participants were invited to a meeting at a local health post, where they were informed about the study’s purpose and procedures. Those who met the inclusion criteria provided written informed consent before enrollment. Recruitment, enrollment, and baseline assessments took place from June to July 2023.

### Sample size and sampling procedure

In this study, 432 couples (432 pregnant women and 432 male partners) participated. The study participants were selected using a cluster sampling technique. Further details on sample size determination and sampling procedures can be found in a previously published study protocol (24).

### Background of the trial

The data source was a baseline survey conducted prior to an ongoing randomized controlled trial intervention aimed at evaluating the effectiveness of couple-based violence prevention education in reducing IPV during pregnancy. The trial is registered on ClinicalTrials.gov under the identifier NCT05856214. The study protocol is published in a peer-reviewed journal (24).

### Ethical approval

The study was approved by Jimma University’s Institutional Review Board (IRB) on November 8, 2022 (JUIH/IRB-222/22). Permission was obtained from the Hadiya Zone Health Department and the districts’ administrative officials. Data collectors explained the study’s purpose and the respondent’s right to refuse participation or answer questions, as detailed in the information sheet. A written informed consent was obtained from all the participants prior to the data collection.

### Baseline survey

In the baseline survey, 432 couples were included from 16 clusters. There were 216 couples in the intervention and 216 control arms, with an average of 27 couples in each cluster. A face-to-face interviewer-administered questionnaire was used to collect the data. We adapted our knowledge questionnaire from studies by Oche et al. (26) and Nmadu et al. (18) conducted in Nigeria, while questionnaires related to attitude toward IPV were adapted from the WHO multi-country study on women’s health and domestic violence against women (27) and Ethiopian Demography and Health Survey (EDHS) 2016 (28). The questionnaires were prepared in English and then translated into an official language (Amharic) and a local language (Hadiyisa). The questionnaires were pre-tested among 5% of the total sample size in Gombora woreda, a district that was not included in our actual study. The interview questionnaire had four parts: socio-demographic and economic parts; reproductive history; questions related to couples knowledge of IPV; and attitudes toward IPV. Wives’ and husbands’ data were gathered independently.

To ensure content validity, subject-matter experts reviewed the questionnaire to evaluate whether the items effectively captured knowledge and attitudes toward IPV. Based on their feedback, modifications were made to improve question clarity and relevance. Face validity was assessed by administering the questionnaire to respondents from a cluster not included in the actual study. Feedback was gathered on the questionnaire’s clarity and comprehension, and necessary revisions were made to enhance its suitability for the study population (29). The internal consistency of the questionnaire was calculated, and Cronbach’s alpha was 0.7 for knowledge and 0.8 for attitude questionnaires, which were acceptable.

### Outcomes of interest and measurement

Knowledge of and attitude toward IPV were the outcomes of the study. The knowledge level of couples was assessed by nine sets of questions. The questions had “yes” or “no “responses, while yes = 1 and no = 0. Couples who scored above the mean score (≥4.5) of the correct answer were considered to have good knowledge, and those below 4.5 were considered to have poor knowledge (18, 26).

A total of five questions were used to assess the attitudes of respondents. Couples were asked whether or not a husband is justified in hitting or beating his spouse in five scenarios: (a) “If she is going out without telling him; (b) “If the wife neglects the children; (c) “If the wife argues with her husband; (d) “If a wife refuses to have sex with her husband; (e) “If the wife burns the food Responses of “supportive attitude toward IPV” to one or more of the scenarios were coded 1; responses of “not supportive attitude toward IPV” to all scenarios were coded “0.” (6).

We used principal component analysis (PCA) to analyze the wealth index of households based on the wealth index questions. Three kinds of household wealth index were produced by the PCA: low, medium, and high economic status.

### Data analysis

We used SPSS version 25.0 to analyze the data. We conducted an analysis stratified by sex. The results of descriptive statistics were presented using texts, graphs, and tables. Bivariable and multivariable logistic regression were used to measure the association between explanatory and outcome variables. A bivariate analysis was conducted to measure the crude odds ratio (COR) with a 95% confidence interval (CI) to assess the association between dependent and independent variables. In the multivariate logistic regression, all variables with a *p*-value <0.25 from the bivariate analysis were included. We verified model fit using the Hosmer and Lemeshow goodness-of-fit test. The strength of the association was determined using the adjusted odds ratio (AOR) and the corresponding 95% confidence interval. All tests were two-tailed, and statistical significance was declared at *p* < 0.05.

## Results

### Socio-demographic and economic characteristics of respondents

A total of 430 out of 432 sampled couples responded to the interview, making a response rate of 99.5%. Two hundred seventy-five (64.0%) of women were in the age group between 25 and 34 years, with a mean age of 29.4 and a standard deviation of 5.7. Two hundred-thirty (53.5%) of the men were in the age group of 25 and 34, with a mean age and standard deviation of 31.4 and 6.5, respectively. Two hundred sixty-one (61.2%) of women and 60.2% of men were protestant Christians, followed by 21.6% of women and 21.6% of men who were Orthodox Christians in their religion. Hadiyas were the main ethnic group, accounting for 64.2% of women and 74.9% of men; 15.3% of women and 13.3% of men were the second most prevalent ethnic group belonging to Kembata.

More than one third, 38.4% of women and 36.0% of men, had not received formal education, while 6.3% of women attended college or higher education and 13.5% of men attended college or higher education. The main occupations were housewives (77.2%) of women and farmers (58.4%) of men. About two-thirds (69.3%) of decisions on household purchases were made by the wife or husband alone, whereas one-third (30.7%) were made by the wife and husband together. Regarding the household wealth index, 33.3% of interviewees had low socioeconomic status, while 33.4 and 33.3% of respondents had medium and high socioeconomic status (Table 1).

**Table 1:** Socio-demographic and economic characteristics of the married couples in Hadiya Zone, Central Ethiopia, July 2023 (*n* = 430).

Characteristics at individual level		Women (*N* = 430) n (%)	Men (*N* = 430) n (%)
Age	15–24	69 (16.0)	55 (12.8)
	25–34	275 (64.0)	230 (53.5)
	35+	86 (20.0)	145 (33.7)
	Mean ± SD	29.4 ± 5.7	31.4 ± 6.5
Mediage at first marriage	<20	83 (19.3)	60 (14.0)
	≥20	347 (80.7)	370 (86.0)
Religion	Protestant Christian	263 (61.2)	259 (60.2)
	Orthodox Christian	93 (21.6)	93 (21.6)
	Others^a^	74 (17.2)	78 (18.2)
Ethnicity	Hadiya	276 (64.2)	322 (74.9)
	Kembata	66 (15.3)	57 (13.3)
	Silte	39 (9.1)	28 (6.5)
	Others^b^	49 (11.4)	23 (5.3)
Education	No education	165 (38.4)	155 (36.0)
	Elementary school	166 (38.6)	137 (31.9)
	Junior or high school	72 (16.7)	80 (18.6)
	College/higher	27 (6.3)	58 (13.5)
Occupation	Housewife	332 (77.2)	251 (58.4)^*^
	Merchant	58 (13.5)	117 (27.2)
	Employed	40 (9.3)	62 (14.4)
Household level characteristics Households (*N* = 430)			
Number of children alive	0–1	66 (15.3)	
	2–4	185 (43.0)	
	5^+^	179 (41.7)	
	Mean ± SD	4.1 ± 2.1	
Household wealth index	Low	143 (33.3)	
	Medium	144 (33.4)	
	High	43 (33.3)	

a Muslim, Catholic, Adventist, Apostolic.

b Amhara, Gurage, Oromo, Wolayita.

* Farmer; NA, Not applicable.

### Reproductive history of women and men’s behavioral characteristics

Of the pregnancies, 33.2% were unintended (20.9% were mistimed, and 12.3% were unwanted). About 42% of the households had five or more living children, while 15.3% of the households had either no children or only one child alive. The mean and standard deviation of the number of living children were 4.1 ± 2.1, with a range of 1 to 10 children alive. Three hundred one (70%) of the pregnant women had received ANC from a skilled health care provider at least once for their recent pregnancy. Out of 301 pregnant women who attended ANC visits, 40.7% were accompanied at least once during ANC visits by their husbands. Men had substance abuse behavior; 27.2% drank alcohol, and 18.2% smoked cigarettes.

### Married couples knowledge of intimate partner violence

The study found that 53.0% [95% CI 48.1–57.7] of women and 58.4% [95% CI 53.5–63.3] of men had good knowledge about IPV. There was no statistically significant difference between women and men in terms of their knowledge (*p* = 0.114). The majority of the respondents, 85.8% of the women and 87.9% of the men, heard of IPV, whereas less than half, 43.0% of women and 47.2% of men, did not know that IPV is a human rights violation (Table 2).

**Table 2:** Responses from married couples to 9 knowledge questions in Hadiya Zone, Central Ethiopia (*n* = 430).

Knowledge questions	Correct response	
	Women N (%)	Men N (%)
Have you heard of intimate partner violence?	369 (85.8)	378 (87.9)
Media, hospitals, and health centers are examples of sources of information for intimate partner violence.	282 (65.6)	261 (60.7)
Intimate partner violence is a serious public health issue.	209 (48.6)	226 (52.6)
Slapping, kicking, dragging, beating, and pushing are examples of physical intimate partner violence.	256 (59.5)	257 (59.8)
Sexual violence occurs when a husband or partner forces the wife or partner to have sex when she does not want to.	195 (45.3)	207 (48.1)
Intimate partner violence occurs when a husband or partner forces his wife or partner to do something sexual that she finds degrading or humiliating.	224 (52.1)	230 (53.5)
Belittling or humiliating a partner in front of other people is psychological violence.	222 (51.6)	234 (55.2)
Intimate partner violence during pregnancy has adverse maternal and newborn outcomes.	202 (47.0)	209 (48.6)
Intimate partner violence is a human rights violation.	185 (43.0)	203 (47.2)

### Married couples attitudes toward intimate partner violence

**Figure 1:**
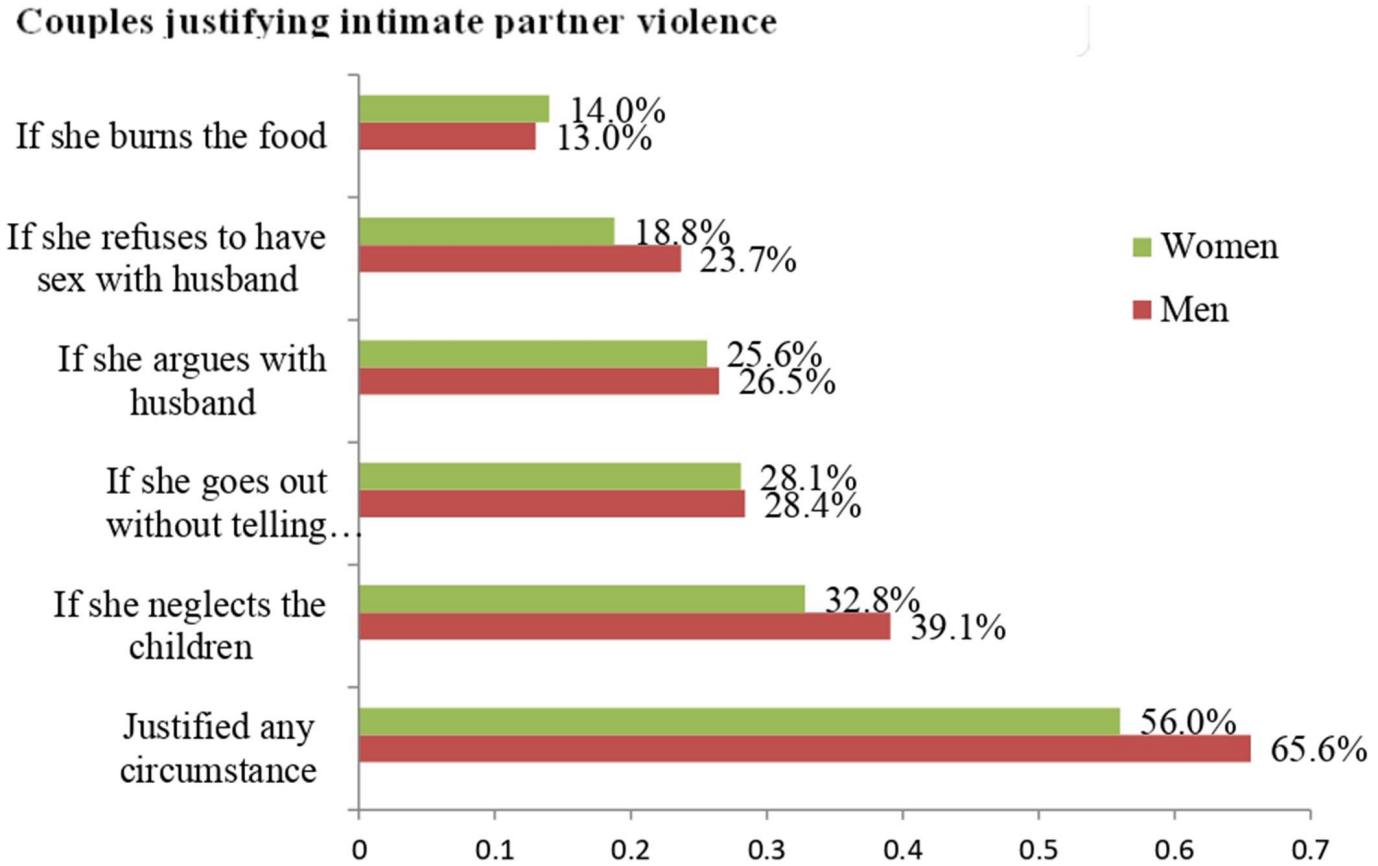
Responses from married couples to five attitude questions in Hadiya Zone, Central Ethiopia (*n* = 430).

In this study, 56.0% [95% CI 51.2–60.9] of women and 65.6% [95% CI 61.2–70.0] of men justified beating their wives or partners in at least one of the five scenarios. There was a statistically significant difference (*p* = 0.005) between the two groups. Meanwhile, 44.0% of women and 34.4% of men agreed that a man could never be justified in beating his wife or spouse. The most frequently mentioned justification for IPV by both men (39.1%) and women (32.8%) was “neglecting the children.” This was followed by “going out without informing the husband” (28.1% of women and 28.4% of men) and “arguing back with the husband” (25.6% of women and 26.5% of men). The scenario least justified for wife/partner beating was ‘burning the food’ with both 14.0% of women and 13.0% of men (Figure 1).

### Factors associated with couples’ knowledge of intimate partner violence

The study found significant associations between women’s knowledge of IPV and age, education, occupation, antenatal care, and their husbands’ alcohol consumption. Similarly, age at marriage, education, marriage type, and alcohol consumption were significantly associated with men’s knowledge of IPV. Women aged 25–34 years were 1.8 times more likely to possess good knowledge of IPV compared to women aged 35 and older [AOR = 1.8; 95%CI: 1.1–3.1]. Men who married before the age of 20 were 50% less likely to have good knowledge compared to the men who married at or after 20 years of age [AOR = 0.5; 95% CI: 0.3–0.9]. Educational status was positively associated with couples’ knowledge of IPV. Women with a high school education were 3.8 times more likely to have good knowledge of IPV compared to those with no education [AOR = 3.8; 95% CI: 2.0–7.3]. Moreover, women with college or higher education were 7.3 times more likely to possess good knowledge of IPV compared to those with no education [AOR = 7.3; 95% CI: 2.3–23.1]. Similarly, men with secondary school education were 2.0 times more likely to have good knowledge of IPV compared to men with no formal education [AOR = 2.0; 95% CI: 1.1–4.1]. Moreover, men with higher or college educations were 2.9 times more likely to have good knowledge of IPV compared to men with no formal education [AOR = 2.9; 95% CI: 1.4–6.0].

Employed women were found to be 3.1 times more likely to have good knowledge of IPV compared to respondents who were housewives [AOR = 3.1; 95% CI: 1.3–7.6]. A significant association was observed between marriage type (monogamy versus polygamy) and knowledge of IPV. Men who had only one wife (monogamy) were 2.6 times more likely to have good knowledge compared to men who had more than one wife (polygamy) [AOR = 2.6; 95% CI: 1.5–4.7]. There was a significant association between women’s knowledge of IPV and ANC during their recent pregnancy. Women who attended ANC during a recent pregnancy were 2.0 times more likely to have good knowledge of IPV compared to women who did not attend ANC [AOR = 2.0; 95% CI: 1.3–3.2]. Alcohol consumption by husbands was negatively associated with couples’ knowledge of IPV. Women with husbands who drink alcohol had 60% lower odds of having good knowledge compared to their counterparts [AOR = 0.4; 95% CI: 0.2–0.7]. Similarly, the odds of having good knowledge were 70% lower among men who drink alcohol [AOR = 0.3; 95% CI: 0.2–0.5] (Table 3).

**Table 3:** Factors associated with couples knowledge of intimate partner violence in Hadiya zone, Central Ethiopia, July 2023 (*N* = 430).

Explanatory variables	Category	Women N (%)	Men N (%)	Adjusted odds ratio (95% CI)	
				Women	Men
Age category	15–24	69 (16.0)	55 (12.8)	1.3 (0.7, 2.6)	0.6 (0.3, 1.3)
	25–34	275 (64.0)	230 (53.5)	1.8 (1.1, 3.1)*	0.9 (0.6, 1.6)
	35+	86 (20.0)	145 (33.7)	1.0	1.0
Age at marriage	<20	83 (19.3)	60 (14.0)	0.7 (0.4, 1.4)	0.5 (0.3, 0.9)*
	≥20	347 (80.7)	370 (86.0)	1.0	1.0
Education	No education	165 (38.4)	155 (36.0)	1.0	1.0
	Elementary school	166 (38.6)	137 (31.9)	1.6 (1.0, 2.5)	1.4 (0.8, 2.3)
	High school	72 (16.7)	80 (18.6)	3.8 (2.0, 7.3)***	1.8 (1.1, 3.5) *
	College/higher	27 (6.3)	58 (13.5)	7.3 (2.3, 23.1)**	2.7 (1.3, 5.4) **
Occupation	Housewife	327 (77.1)	251 (58.4)^a^	1.0	0.9 (0.5, 1.7)
	Merchant	58 (13.7)	117 (27.2)	1.0 (0.5, 1.9)	1.7 (0.8, 3.5)
	Employed	39 (9.2)	62 (14.4)	3.1 (1.3, 7.6)*	1.0
Marriage type	Monogamy	357 (83.0)	357 (83.0)	NI	2.6 (1.5, 4.7)**
	Polygamy	73 (17.0)	73 (17.0)	NI	1.0
ANC	Yes	299 (70.5)	NA	2.0 (1.3, 3.2)**	NA
	No	125 (29.5)	NA	1.0	NA
Husband smokes cigarette	Yes	81 (18.2)	81 (18.2)	0.7 (0.4, 1.3)	1.0 (0.5, 1.8)
	No	349 (81.8)	349 (81.8)	1.0	1.0
Husband drinks alcohol	Yes	117 (27.2)	117 (27.2)	0.4 (0.3, 0.7)**	0.3 (0.2, 0.5) ***
	No	313 (72.8)	313 (72.8)	1.0	1.0
No of living children	0–1	66 (15.3)	66 (15.3)	1.1 (0.6, 2.3)	0.9 (0.5, 1.7)
	2–4	185 (43.0)	185 (43.0)	1.0 (0.6, 1.7)	1.3 (0.8, 2.1)
	5^+^	179 (41.7)	179 (41.7)	1.0	1.0
Household wealth index	Low	141 (33.3)	141 (33.3)	0.9 (0.6, 1.6)	0.8 (0.5, 1.4)
	Medium	142 (33.4)	142 (33.4)	1.5 (0.9, 2.5)	0.6 (0.4, 1.0)
	High	141 (33.3)	141 (33.3)	1.0	1.0

a Farmer; ***p < 0.001, **p < 0.01, *p < 0.05; IPV, Intimate Partner Violence; ANC, Antenatal Care; Not Included in the multivariate model; 1.0 = Reference category.

### Factors associated with couples’ attitudes toward intimate partner violence

Women’s attitudes toward IPV were significantly associated with their age at marriage, educational level, husband’s cigarette smoking, and knowledge of IPV. In addition, age, polygamy, alcohol consumption, and knowledge of IPV were found to be predictors of men’s attitudes toward IPV. Women who married before the age of 20 were about 2.0 times more likely to have a supportive attitude toward IPV [AOR = 1.9; 95% CI: 1.1–3.3]. Men aged 15–24 years were 5.8 times more likely to possess a supportive attitude toward IPV compared to men aged 35 and older [AOR = 5.8; 95%CI: 2.2–14.9].

Educational attainment was negatively associated with attitudes toward IPV. Women with a high school education were found to be 60% less likely to have good knowledge of IPV compared to those with no education [AOR = 0.4; 95% CI: 0.2–0.8]. Moreover, women with higher or college education were 80% less likely to have good knowledge of IPV compared to those with no education [AOR = 0.2; 95% CI: 0.1–0.5]. The odds of having a supportive attitude toward IPV were 1.9 times higher among women whose husbands smoked cigarettes compared to women whose husbands did not smoke [AOR = 1.9; 95% CI: 1.1–3.5]. Men who had only one wife (monogamy) were 60% less likely to have a supportive attitude toward IPV compared to men who had more than one wife (polygamy) [AOR =0.4; 95% CI: 0.2–0.9]. Alcohol consumption was associated with a 3.0-fold increase in the odds of men having a supportive attitude toward IPV [AOR = 3.0; 95% CI: 1.7–5.4]. Knowledge of IPV was significantly associated with couples’ attitudes toward IPV. Women who had poor knowledge of IPV were 1.9 times more likely to have a supportive attitude toward IPV compared to women who had good knowledge of IPV [AOR = 1.9; 95% CI: 1.3–2.9]. Similarly, men who had poor knowledge of IPV were 2.4 times more likely to possess a supportive attitude toward IPV compared to their counterparts (Table 4).

**Table 4:** Factors associated with couples attitude toward intimate partner violence in Central Ethiopia, July 2023 (*N* = 430).

Explanatory variables	Category	Women N (%)	Men N (%)	Adjusted odds ratio (95% CI)	
				Women	Men
Age category	15–24	69 (16.0)	55 (12.8)	0.8 (0.3, 1.9)	5.8 (2.2, 14.9)***
	25–34	275 (64.0)	230 (53.5)	1.0 (0.6, 1.8)	1.4 (0.9, 2.3)
	35+	86 (20.0)	145 (33.7)	1.0	1.0
Age at marriage	<20	83 (19.3)	60 (14.0)	1.9 (1.1, 3.3)*	1.1 (0.5, 2.3)
	≥20	347 (80.7)	370 (86.0)	1.0	1.0
Education	No education	165 (38.4)	155 (36.0)	1.0	1.0
	Elementary school	166 (38.6)	137 (31.9)	1.0 (0.7, 1.7)	0.8 (5, 1.5)
	High school	72 (16.7)	80 (18.6)	0.4 (0.2, 0.8)**	0.6 (0.3, 1.1)
	College/higher	27 (6.3)	58 (13.5)	0.2 (0.1, 0.5)**	0.7 (0.4, 1.5)
Occupation	Housewife	327 (77.1)	251 (58.4)^a^	1.7 (0.8, 3.6)	1.5 (0.8, 2.8)^a^
	Merchant	58 (13.7)	117 (27.2)	1.3 (0.5, 3.2)	0.7 (0.4, 1.5)
	Employed	39 (9.2)	62 (14.4)	1.0	1.0
Marriage type	Monogamy	357 (83.0)	357 (83.0)	NI	0.4 (0.2, 0.9)*
	Polygamy	73 (17.0)	73 (17.0)	NI	1.0
Husband smokes cigarrete	Yes	299 (70.5)	299 (70.5)	1.9 (1.1, 3.5)*	0.9 (0.5, 1.7)
	No	125 (29.5)	125 (29.5)	1.0	1.0
Drink alcohol	Yes	81 (18.2)	81 (18.2)	1.2 (0.7, 2.1)	3.0 (1.7, 5.4)***
	No	349 (81.8)	349 (81.8)	1.0	1.0
Number of living children	0–1	117 (27.2)	117 (27.2)	1.2 (0.6, 2.4)	1.0 (0.5, 1.9)
	2–4	313 (72.8)	313 (72.8)	0.9 (0.6, 1.5)	0.6 (0.4, 1.0)
	5^+^	66 (15.6)	66 (15.6)	1.0	1.0
Knowledge of IPV	Good	228 (53.0)	251 (58.4)	1.0	1.0
	Poor	202 (47.0)	179 (41.6)	1.9 (1.3, 2.9)**	2.4 (1.5, 3.9)***
Household wealth index	Low	143 (33.3)	143 (33.3)	1.3 (0.8, 2.1)	0.6 (0.3, 1.0)
	Medium	144 (33.4)	144 (33.4)	1.2 (0.7, 2.0)	0.8 (0.5, 1.4)
	High	143 (33.3)	143 (33.3)	1.0	1.0

a Farmer; ***p < 0.001, **p < 0.01, *p < 0.05; AOR, Adjusted Odds Ratio; NA, Not Applicable; NI, not included in the model; 1.0 = Reference category.

## Discussion

This study found that the level of awareness regarding IPV among women and men revealed intriguing disparities. Notably, 53.0% of women had good knowledge of IPV, a figure akin to findings in a comparable study in Kaduna, Nigeria (66.5%) (18). However, this percentage falls short in comparison to a study conducted in Sokoto, Nigeria, where a remarkable 99.2% of participants had good knowledge of IPV (26). Several factors could contribute to these disparities, including differences in educational attainment, study settings, cultural norms, and societal attitudes toward discussing or acknowledging IPV. In this study, it’s noteworthy that over one-third (36.1%) of respondents lacked formal education, and participants were exclusively from rural areas, potentially limiting their exposure to information about IPV. The disparity between the present study and Nigerians could be, for example, that the study in Sokoto, Nigeria, included a significant proportion of university or diploma graduates (36.4%), with a majority residing in urban areas (72.7%) (26).

The study also revealed men’s levels of knowledge of IPV. More than half (58.4%) of the men included in the study had good knowledge of IPV. However, because there has been no study on men’s knowledge of IPV, it is impossible to compare our findings to those of other studies. This emphasizes the significance of conducting more research to assess men’s overall knowledge of IPV.

In the current study, 56% of women expressed their supportive attitude toward IPV in at least one of the five scenarios. This is consistent with finding Arbaminch, Ethiopia (59.5%) (30). In contrast with evidence from a study across 30 countries in Sub-Saharan Africa (7), in our study, men (65.6%) had slightly higher levels of supportive attitudes toward IPV than women (56.0%).

This study found that men (65.6%) exhibited slightly higher levels of supportive attitudes toward IPV compared to women (56.0%). This result is consistent with findings from rural India (31) and a comparative analysis of 17 Sub-Saharan countries (32), which revealed that in Lesotho, men were significantly more likely than women to accept IPV. However, this contrasts with other studies (7, 33), where women demonstrated more supportive attitudes toward IPV under certain circumstances, such as neglecting children, going out without informing the husband, arguing back with the husband, and refusing sex with the husband. Our findings suggest that men deeply ingrain traditional gender norms and patriarchal values. The prevalence of supportive attitudes among men in our study was significantly higher than the findings of other Sub-Saharan African countries such as Ghana (34%) (34), Zambia (35.7%) (47), and Zimbabwe (24.4%) (47). This disparity could be attributed to differences in cultural norms, gender roles within society and the study participants’ educational levels, with one-third (36.0%) lacking formal education in our study. This finding underscores the importance of interventions addressing societal attitudes and beliefs toward IPV.

The study identified various factors influencing couples’ knowledge of IPV. Women aged 25–34 were about two times more likely to have good knowledge of IPV compared to women aged 35 and older. This might be because women in the middle age group may have a better educational status and greater access to information compared to older women. This finding is in line with the study findings in Nigeria (18). Men who married before the age of 20 were 50% less likely to have good knowledge compared to the men who married at or after 20 years of age. This could be the fact that those who marry early may spend less time in formal education and may have fewer opportunities to access information about IPV.

In this study, educational attainment was found to be a significant predictor of IPV knowledge among couples. Couples with high school and college/higher education were more likely to have good knowledge of IPV compared to those couples with no formal education. This finding emphasizes the transformative power of education in equipping individuals with knowledge and resources, enabling them to engage with sensitive topics such as IPV through formal education and self-directed learning (35). This finding implies that interventions targeting couples with no formal education can help mitigate IPV.

Moreover, the study found that men’s employment status was significantly associated with their knowledge of IPV. Employed women were nearly three times more likely to be knowledgeable about IPV than housewives, which is consistent with prior research findings in Sri Lanka (19). This could be due to the fact that employed women might have access to IPV-related information. Antenatal care visits during a recent pregnancy were found to be a significant predictor of knowledge of IPV. These insights highlight the crucial role of ANC visits as a platform for disseminating information and resources about IPV, thereby enhancing awareness among pregnant women (2). Moreover, marriage type was emerged as a significant determinant, with men in monogamous marriages showing a 2.4 times higher likelihood of having good knowledge of IPV compared to those in polygamous marriages. This disparity may be attributed to several factors, including the fact that men in polygamous marriages may have limited opportunities for information exchange within marital relationships and reduced time available for discussing sensitive topics like IPV with multiple spouses (36).

Interestingly, this study found the influence of husbands’ alcohol consumption on couples’ knowledge of IPV. Women with alcohol-consuming spouses were 60% less likely to have good knowledge of IPV. Similarly, men who consume alcohol were 70% less likely to possess good knowledge of IPV. This could be because alcohol consumption impairs effective communication within the marital partnership, preventing discussions about sensitive topics such as IPV and limiting the couples’ access to information and resources. This is consistent with the previous literature, which shows the deleterious effects of alcohol usage on interpersonal dynamics within intimate relationships (12, 37). This finding accentuates the critical importance of targeted educational interventions tailored to reach men who consume alcohol, highlighting the potential barriers posed by substance use in accessing and assimilating information about IPV.

The study also uncovered significant associations between various factors and couples’ attitudes toward IPV. Significantly, women who married before the age of 20 had approximately twice the odds of having supportive attitudes toward IPV compared to their counterparts. This finding aligns with previous research conducted in Bangladesh (38), Pakistan (23), and Ethiopia (30), where younger women were more likely to accept IPV under certain circumstances. The study also revealed that men in the younger age group (6–15) were 5.8 times more likely to have a supportive attitude toward IPV compared to their counterparts in the older age group (35 and above). This finding was echoed in corroborative evidence from the National Family Health Survey of India (20), a study in Indonesia (48), and evidence from 30 countries in Sub-Saharan Africa (7). Across these diverse contexts, it consistently appeared that older men exhibited a diminished inclination to condone IPV when compared with their younger counterparts. The plausible explanations could be that the relative lack of life experience and exposure to the detrimental consequences of IPV among younger men could contribute to their higher tolerance levels. Moreover, societal normalization of relationship violence coupled with deficient interpersonal dynamics and communication skills might further perpetuate such attitudes, particularly among younger demographics (49).

Educational attainment was found to be a significant predictor of women’s attitudes toward IPV. Women having with secondary, tertiary, or college education levels had a decreased likelihood of having supportive attitudes compared to those with no formal education. This finding corroborates existing literature, highlighting the consistent trend of education serving as a protective factor against supportive attitudes toward IPV (19, 21, 23). Consistent with the previous evidence, it was found that men in monogamous marriages were 60% less likely to have a supportive attitude toward IPV compared to men in polygamous marriages (48, 50). This discrepancy could potentially be attributed to the cultural norms prevalent in monogamous marriages, which often prioritize principles of mutual respect and equality within marital dynamics. Conversely, within polygamous settings, where power differentials are more pronounced and hierarchical structures prevail, violence may be perceived as a tool for asserting dominance and control over spouses, thereby fostering a more permissive attitude toward IPV (36).

Furthermore, the study investigated the association between women’s attitudes toward IPV and their husbands’ smoking habits, revealing that women with smoking husbands were four times more likely to have supportive attitudes toward IPV compared to those with non-smoking husbands. This phenomenon may be attributed to increased marital conflict experienced by women with smoking husbands, potentially leading to the normalization of violence as a means of conflict resolution. Men’s alcohol consumption was found to be the predictor of attitudes toward IPV. Men who consume alcohol were three times more likely to have a supportive attitude toward IPV. In line with this result, evidence from the National Family Health Survey of India has found that men who drink alcohol are more likely to accept wife beating in certain circumstances compared to those who do not drink alcohol (20). This could be because alcohol consumption can hinder cognitive functions and decision-making, leading to aggressive or dismissive attitudes toward wives and justifying violence as a means to resolve conflicts or exert control.

Additionally, the study found that poor knowledge of IPV was significantly associated with supportive attitudes toward IPV, with women lacking good knowledge demonstrating 2.3 times higher odds of having supportive attitudes. Similarly, men’s poor knowledge of IPV increased the odds of a supportive attitude toward IPV by 2.4 times. Couples with limited knowledge about IPV may have preconceptions and views that encourage supportive attitudes. Targeted educational programs addressing IPV, its health, social, and economic impact, challenging misconceptions, and promoting healthy relationships are important in changing attitudes toward IPV (51). This underscores the importance of addressing knowledge gaps regarding IPV, as limited understanding may lead to the normalization of abusive behaviors within relationships (52). Interventions targeting both knowledge enhancement and attitude transformation might help address a comprehensive IPV prevention and control efforts (26).

This study’s findings have substantial implications for developing prevention interventions and comprehensive support systems for IPV survivors in rural Ethiopia. The identified factors, such as educational attainment, attitudes toward IPV, and younger age, provide a foundation for designing culturally tailored interventions that prioritize awareness and mitigate supportive attitudes toward IPV within the community. Additionally, the role of male alcohol consumption and smoking as predictors of IPV-supportive attitudes highlights the need for interventions that address substance abuse as part of IPV prevention and treatment programs.

This study had some limitations. Due to the cross-sectional nature of the study design, it was not possible to establish a causal relationship between explanatory and outcome variables. Social desirability bias poses a potential concern, wherein respondents may provide answers that align with societal or researcher expectations regarding attitudes toward IPV. However, gender-matched field supervisors and data collectors were used, who underwent extensive training prior to conducting fieldwork. Another limitation was the scarcity of literature on men’s knowledge and attitudes toward IPV, limiting our ability to compare our results. This scarcity underscores a necessitating further study to gain a more comprehensive understanding of knowledge levels, attitudes, and associated factors in diverse settings.

## Conclusion

The study resulted in insightful findings regarding the knowledge and attitudes toward IPV among women and men. Among women, 53.0% demonstrated good knowledge of IPV, while 56.0% had supportive attitudes. For men, 58.4% had good knowledge, and 65.6% expressed supportive attitudes toward IPV. The study identified significant correlations with age (25–34 years), educational attainment (secondary and higher/college education), attendance at ANC visits, and husbands’ alcohol consumption. Younger age, secondary and higher/college education, monogamous marriages, and alcohol consumption were found to be predictors of men’s knowledge. The study also found that early marriage (below 20 years), educational attainment of secondary and higher/college education, partner smoking, and poor knowledge of IPV were predictors of women’s attitude toward IPV. Also, younger age, monogamous marriage, alcohol consumption, and poor knowledge of IPV were found to be predictors of men’s attitudes toward IPV.

The findings imply the need for specific interventions. Decision-makers at different levels should prioritize improving women’s education, challenging societal attitudes, promoting ANC visits, addressing male substance abuse, and increasing couples’ understanding and awareness of IPV and its consequences.

## Data Availability

The raw data supporting the conclusions of this article will be made available by the authors, without undue reservation.
